# Association of Dual Decline in Memory and Gait Speed With Risk for Dementia Among Adults Older Than 60 Years: A Multicohort Individual-Level Meta-analysis

**DOI:** 10.1001/jamanetworkopen.2019.21636

**Published:** 2020-02-21

**Authors:** Qu Tian, Susan M. Resnick, Michelle M. Mielke, Kristine Yaffe, Lenore J. Launer, Palmi V. Jonsson, Giulia Grande, Anna-Karin Welmer, Erika J. Laukka, Stefania Bandinelli, Antonio Cherubini, Caterina Rosano, Stephen B. Kritchevsky, Eleanor M. Simonsick, Stephanie A. Studenski, Luigi Ferrucci

**Affiliations:** 1 Longitudinal Studies Section, Translational Gerontology Branch, National Institute on Aging, Baltimore, Maryland; 2 Laboratory of Behavioral Neuroscience, National Institute on Aging, Baltimore, Maryland; 3 Division of Epidemiology, Department of Health Sciences Research, Mayo Clinic, Rochester, Minnesota; 4 Department of Neurology, Mayo Clinic, Rochester, Minnesota; 5 Department of Psychiatry, University of California, San Francisco; 6 Department of Neurology, University of California, San Francisco; 7 Department of Epidemiology, University of California, San Francisco; 8 Laboratory of Epidemiology and Population Studies, National Institute on Aging, National Institutes of Health, Baltimore, Maryland; 9 Faculty of Medicine, University of Iceland, Reykjavik, Iceland; 10 Karolinska Institutet, Aging Research Center, Department of Neurobiology, Care Sciences and Society, Stockholm University, Stockholm, Sweden; 11 Division of Physiotherapy, Department of Neurobiology, Care Sciences and Society, Karolinska Institutet, Stockholm, Sweden; 12 Karolinska University Hospital, Stockholm, Sweden; 13 Stockholm Gerontology Research Center, Stockholm, Sweden; 14 Geriatric Unit, Azienda Sanitaria di Firenze, Florence, Italy; 15 Geriatria, Accettazione Geriatrica e Centro di ricerca per l’invecchiamento, IRCCS Istituto Nazionale Ricovero e Cura per Anziani, Ancona, Italy; 16 Department of Epidemiology, Graduate School of Public Health, University of Pittsburgh, Pittsburgh, Pennsylvania; 17 The Sticht Center for Healthy Aging and Alzheimer’s Prevention, Wake Forest School of Medicine, Winston-Salem, North Carolina

## Abstract

**Importance:**

Dual decline in both memory and gait speed may characterize a group of older individuals at high risk for future dementia.

**Objective:**

To assess the risk of dementia in older persons who experience parallel declines in memory and gait speed compared with those who experience no decline or decline in either memory or gait speed only.

**Design, Setting, and Participants:**

A multicohort meta-analysis was performed of 6 prospective cohort studies conducted between 1997 and 2018 in the United States and Europe. Participants were 60 years or older, had an initial gait speed of more than 0.6 m/s (ie, free of overt dismobility), with repeated measures of memory and gait speed before dementia diagnosis during a mean follow-up of 6.6 to 14.5 years. Within each study, participants were divided into 4 groups: memory decline only, gait speed decline only, dual decline, or no decline (hereafter referred to as *usual agers*). Gait decline was defined as a loss of 0.05 m/s or more per year; memory decline was defined as being in the cohort-specific lowest tertile of annualized change.

**Main Outcomes and Measures:**

Risk of incident dementia according to group membership was examined by Cox proportional hazards regression with usual agers as the reference, adjusted for baseline age, sex, race/ethnicity, educational level, study site, and baseline gait speed and memory.

**Results:**

Across the 6 studies of 8699 participants, mean age ranged between 70 and 74 years and mean gait speed ranged between 1.05 and 1.26 m/s. Incident dementia ranged from 5 to 21 per 1000 person-years. Compared with usual agers, participants with only memory decline had 2.2 to 4.6 times higher risk for developing dementia (pooled hazard ratio, 3.45 [95% CI, 2.45-4.86]). Those with only gait decline had 2.1 to 3.6 times higher risk (pooled hazard ratio, 2.24 [95% CI, 1.62-3.09]). Those with dual decline had 5.2 to 11.7 times the risk (pooled hazard ratio, 6.28 [95% CI, 4.56-8.64]).

**Conclusions and Relevance:**

In this study, dual decline of memory and gait speed was associated with increased risk of developing dementia among older individuals, which might be a potentially valuable group for preventive or therapeutic interventions. Why dual decline is associated with an elevated risk of dementia and whether these individuals progress to dementia through specific mechanisms should be investigated by future studies.

## Key Points

**Question:**

Is a decline in both memory and gait speed with aging associated with a higher risk of dementia than no decline or a decline in memory or gait only in older adults?

**Findings:**

In this meta-analysis of 6 studies including 8699 participants from the United States and Europe, a decline in both memory and gait was associated with 6.28 times higher risk of developing dementia than no decline.

**Meaning:**

Older adults without dementia with parallel declines in memory and gait are associated with high risk of developing dementia and may be a group to target for prevention.

## Teaser

This meta-analysis assesses whether parallel declines in memory and gait speed among older adults, compared with those who experience no decline or decline in either memory or gait speed only, are associated with risk of developing dementia.

## Introduction

Impaired mobility, such as slow gait, is associated with an increased risk of dementia, but the effect size of this association is generally modest.^1,2,3,4,5,6^ Identifying persons who experience both mobility decline and memory decline, a main symptom in the early stage of dementia, may have a greater prognostic value in assessing risk of dementia because the combination could identify a group in whom gait speed decline is at least in part caused by neurodegenerative pathologic conditions of the central nervous system rather than local musculoskeletal problems, such as sarcopenia or osteoarthritis.^7,8,9^ A recent study of 154 participants with mild cognitive impairment reported that those who declined in both cognition and gait speed had the highest risk of dementia.^10^ However, this study population was limited to a relatively small clinical sample admitted to geriatric clinics. Whether this association occurs in general aging populations initially free of dismobility and cognitive impairment is unknown. If such an association is confirmed, it may help to identify older individuals who are free of dismobility and cognitive impairment but at high risk of developing dementia. It could also motivate investigations into whether this group develops dementia through specific pathophysiological mechanisms.

A growing line of research suggests that motoric cognitive risk syndrome, a combination of cognitive decline, slow gait, and preserved activities of daily living, is associated with high risks of cognitive impairment and dementia.^11,12,13,14^ However, motoric cognitive risk syndrome is typically operationalized at 1 time point. Recent evidence suggests that assessing gait speed longitudinally rather than with a 1-time assessment better distinguishes individuals who develop dementia.^10^

To address these knowledge gaps, we examined whether the extreme phenotype of dual decline in memory and gait with aging compared with no decline or decline in gait or memory only would be associated with an increased risk of dementia among community-dwelling older adults. We used data from 6 prospective cohort studies in the United States and Europe to create an individual-level meta-analysis. To capture early memory and gait changes with aging, we identified participants aged 60 years or older who were initially free of dismobility, cognitive impairment, and dementia; tracked changes in memory and gait speed during the dementia-free period; and then captured incident dementia. We hypothesized that those who experienced a decline in both memory and gait speed would have a higher risk of dementia than those with no decline or with a decline in gait or memory only.

## Methods

### Study Population

We used individual-level data from 6 prospective cohort studies: the Baltimore Longitudinal Study of Aging (BLSA)^15^; the Health, Aging and Body Composition Study (Health ABC)^16^; the Mayo Clinic Study of Aging (MCSA)^17^; the Age, Gene/Environment Susceptibility-Reykjavik Study (AGES-RS)^18^; the InCHIANTI (Invecchiare in Chianti, Aging in the Chianti Area) study^19^; and the Swedish National Study on Aging and Care-Kungsholmen Population Study (SNAC-K).^20^ The years of data collection ranged between 1997 and 2018. All studies were conducted under the oversight and approval of the institutional review boards of the institutions that conducted them. All participants consented to participate after receiving a comprehensive description of the study, including possible risks. This meta-analysis followed the Preferred Reporting Items for Systematic Reviews and Meta-analyses (PRISMA) reporting guideline.

We excluded participants who, at baseline, had prevalent cognitive impairment, dementia, and dismobility (gait speed ≤0.6 m/s)^21^ and who had baseline Mini-Mental State Examination (MMSE) scores of less than 24^22^ (BLSA, MCSA, AGES-RS, InCHIANTI, and SNAC-K) or Modified Mini-Mental State Examination scores of less than 80 (Health ABC).^23^ Mild cognitive impairment or cognitive impairment was diagnosed by consensus conferences following published criteria in BLSA,^24^ MCSA,^25^ AGES-RS,^26^ and InCHIANTI.^19^ We further excluded participants who did not have repeated measures of memory and gait speed prior to diagnoses of dementia, last follow-up, or death because, for these individuals, we could not estimate annualized changes in memory and gait speed.

### Phenotypic Groups

Within each study, participants were classified into 4 phenotypic groups based on annualized changes in memory and gait speed prior to diagnoses of dementia, last follow-up, or death. Usual gait speed for a distance of 7.6 m (25 ft) in MCSA or a distance of 6 m in all other studies was used for analysis. Gait speed was harmonized to the same unit in meters per second. Specifically, in InCHIANTI, we converted a 4-m gait speed to a 6-m gait speed.^27^ In Health ABC, we converted a 20-m gait speed assessed at years 2, 3, 5, and 8 to a 6-m gait speed.^28^ In MCSA, gait speed was simply expressed as meters (25 ft; 7.62 m) divided by time in seconds.^29^ Except in MCSA and SNAC-K, which conducted only 1 trial, usual gait speed was assessed in 2 trials, and the faster trial was used for analysis.

Across the 6 studies, we focused on verbal episodic memory when available, especially immediate recall, because it was among the first cognitive functions to decline among Alzheimer disease–related cognitive measures.^30^ Specifically, memory was assessed using the California Verbal Learning Test in BLSA,^31^ the Buschke Selective Reminding Test in Health ABC,^32^ the Rey Verbal Learning Test in MCSA,^17,33^ the modified California Verbal Learning Test in AGES-RS,^31^ the MMSE memory subscore in InCHIANTI,^34^ and a free recall test in SNAC-K.^35^

### Outcome

Dementia was ascertained based on the *Diagnostic and Statistical Manual of Mental Disorders* (Third Edition Revised)^36^ in BLSA; the *Diagnostic and Statistical Manual of Mental Disorders* (Fourth Edition) (*DSM-IV*) in MCSA and SNAC-K; race-specific decline in Modified Mini-Mental State Examination scores, medication use, and hospital records in Health ABC^37^; a 3-step consensus adjudication following *DSM-IV* criteria in AGES-RS^18^; and a 2-stage screening procedure following *DSM-IV* criteria in InCHIANTI.^38^ Within each study, the end point was at diagnoses of dementia for those who developed dementia or at last follow-up or death for those who did not develop dementia. Detailed assessment and study designs are presented in eTable 1 in the Supplement.

### Statistical Analysis

Within each study, annual rates of change in memory and gait speed prior to diagnoses of dementia, last follow-up, or death were first computed using simple linear regression. Participants were then classified into 4 phenotypic groups, using cut points of annualized decline in gait speed equal to or greater than 0.05 m/s^39^ and the lowest tertile of annualized decline in memory performance. Specifically, those with less than 0.05 m/s annualized decline in gait speed and in the middle and highest tertiles (ie, less decline) of annualized decline in memory were referred to as *usual agers*. Those with less than 0.05 m/s annualized decline in gait speed but in the lowest tertile of annualized decline in memory were referred to as participants with memory decline only. Those with equal to or greater than 0.05 m/s annualized decline in gait speed and in the middle and highest tertile of annualized decline in memory were referred to as participants with gait decline only. Those with equal to or greater than 0.05 m/s annualized decline in gait speed and in the lowest tertile of annualized decline in memory were referred to as participants with dual decline. In InCHIANTI, individual slopes of MMSE memory subscores were estimated using a random-effects Poisson model owing to its distribution.

We first examined whether baseline gait speed and memory differed between groups, using multiple linear regression with usual agers being the reference group, adjusting for baseline age, sex, educational level, race/ethnicity, and study site. We then examined associations of baseline gait speed and memory performance with incident dementia using Cox proportional hazards regression, accounting for demographic characteristics.

We examined associations of phenotypic groups with incident dementia using Cox proportional hazards regression, with usual agers being the reference group, adjusting for demographic characteristics and baseline gait speed and memory performance. In Health ABC, because Buschke Selective Reminding Test began at year 3, the estimated intercept at baseline by simple linear regression was used as the covariate.

The mean risk of dementia among participants with memory decline, participants with gait decline, and participants with dual decline across the 6 studies was examined using random-effects meta-analysis, following guidelines from the *Cochrane Handbook for Systematic Reviews of Interventions*.^40^ The findings are presented as forest plots and reported following published guidelines.^41^ Heterogeneity among studies was examined using the Cochrane χ^2^ test. The amount of variation across studies that was due to heterogeneity was indicated by *I*^2^, expressed as a percentage. We performed sensitivity analyses by excluding 2 studies in which dementia was ascertained a posteriori (Health ABC and InCHIANTI). We performed additional sensitivity analyses by adjusting for baseline multimorbidity and specific diseases important for declines in memory and gait, including hypertension, cardiovascular disease, and stroke.

To evaluate whether changes in gait speed and memory added prognostic value beyond baseline gait speed and memory, we compared 2 models using likelihood ratio tests. To examine whether the risk of dementia associated with gait decline was similar for those with and those without memory decline, we added an interaction term between slope of memory decline and slope of gait decline in Cox proportional hazards regression. A significant interaction would reject the null hypothesis that the risk of developing dementia associated with gait decline was the same for participants with and participants without memory decline.

Statistical analyses were performed using SAS, version 9.4 (SAS Institute Inc) and R, version 3.5.0 (R Project for Statistical Computing). All *P* values were from 2-sided tests and results were deemed statistically significant at *P* < .05.

## Results

All 6 studies of 8699 participants included both men and women (Table 1). The BLSA and Health ABC study populations were racially/ethnically diverse and included a substantial proportion of black participants. Other studies had almost all or exclusively white participants. At baseline, the mean age across studies ranged between 70 and 74 years. The mean gait speed ranged between 1.05 and 1.26 m/s. The mean follow-up time ranged between 6.6 and 14.5 years. Across the 6 studies of 8699 participants, incident dementia ranged from 5 to 21 per 1000 person-years.

**Table 1.** Baseline Sample Characteristics

Characteristic	BLSA (N = 664)	Health ABC (N = 727)	MCSA (N = 2633)	AGES-RS (N = 2563)	InCHIANTI (N = 553)	SNAC-K (N = 1559)
Study entry	2006^a^	1997/1998	2004	2002	1998	2001-2004
Study site	Baltimore, MD	Pittsburgh, PA; Memphis, TN	Olmsted County, MN	Iceland	Greve in Chianti, Bagno a Ripoli, Italy	Stockholm, Sweden
Demographic						
Age, mean (SD) [range], y	73.3 (8.2) [60-95]	73.5 (2.7) [69-81]	74.3 (7.0) [60-91]	74.4 (4.5) [66-91]	71.8 (5.3) [65-91]	70.4 (8.9) [60-97]
Women, No. (%)	329 (50)	371 (51)	1266 (48)	1491 (58)	293 (53)	947 (61)
Black race/ethnicity, No. (%)	140 (21)	315 (43)	5 (0.2)	0	0	0
Educational level, mean (SD) [range], y	17.6 (2.7) [7-32]	13.7 (2.8) [3-18]	14.5 (2.7) [5-20]	No. (%), 783 (31) >high school	6.3 (3.4) [0-22]	No. (%), 658 (42) >high school
Body mass index, mean (SD) [range]^b^	26.8 (4.4) [17.8-45.6]	27.0 (4.5) [15.6-48.0]	28.3 (5.2) [16.9-57.8]	27.3 (4.1) [15.6-47.5]	27.4 (3.9) [18-47]	26.0 (3.7) [16-47]
Global mental status^c^	MMSE	3MS	MMSE	MMSE	MMSE	MMSE
Median (IQR) score [range]	29 (28-30) [24-30]	94 (90-97) [80-100]	28 (27-29) [24-30]	28 (27-29) [24-30]	27 (26-28) [24-30]	29 (29-30) [25-30]
Memory performance^c^	CVLT	SRT	AVLT	Modified CVLT	MMSE memory subscore	Word recall
Mean (SD) score [range]	51.0 (12.0) [6-80]	46.8 (11.3) [2-72]	40.7 (9.3) [13-69]	28.6 (7.1) [0-57]	5 (4-6) [3-6]^d^	7.5 (2.3) [0-16]
Gait speed, m/s^e^						
Mean (SD) speed [range]	1.16 (0.21) [0.61-1.97]	1.26 (0.24) [0.69-2.0]	1.12 (0.23) [0.64-1.91]	1.05 (0.18) [0.61-1.74]	1.19 (0.22) [0.62-2.0]	1.21 (0.29) [0.61-2.00]
Incident dementia						
No. (%)	45 (7)	130 (18)	92 (3)	254 (10)	123 (22)	165 (11)
Per 1000 person-years	8	14	5	9	21	10
No. of visits, mean (SD) [range]^f^	4.2 (1.8) [2-12]	8 (1) [3-9]	4.5 (2.1) [2-11]	2 (0)^g^	3.6 (1.1) [2-5]	3 (1) [2-5]
Follow-up time, mean (SD) [range], y	8.3 (2.8) [2.0-13.1]	12.2 (3.0) [3.9-17.1]	6.6 (2.9) [1.3-13.0]	10.7 (1.6) [4.7-13.0]	14.5 (4.4) [3-20]	10.0 (2.7) [2-14]
No. of visits per year, mean (SD) [range]	1 (0.3) [0.2-2.0]	1 (0.1) [0.5-1.5]	1 (0.3) [0.2-2.2]	0.4 (0.02) [0.2-0.8]	0.5 (0.1) [0.2-0.7]	0.3 (0.1) [0.1-1.0]
Phenotypic groups, No. (%)						
No memory or gait speed decline	375 (56)	422 (58)	1383 (53)	1519 (59)	291 (53)	674 (43)
Memory decline only	170 (26)	202 (28)	616 (23)	743 (29)	164 (30)	309 (20)
Gait decline only	67 (10)	63 (9)	373 (14)	177 (7)	61 (11)	365 (23)
Dual decline in memory and gait speed	52 (8)	40 (6)	261 (10)	124 (5)	37 (7)	211 (14)

Abbreviations: 3MS, Modified Mini-Mental State Examination; AGES-RS, Age, Gene/Environment Susceptibility-Reykjavik Study; AVLT, Auditory Verbal Learning Test; BLSA, Baltimore Longitudinal Study of Aging; CVLT, California Verbal Learning Test; Health ABC, Health, Aging and Body Composition Study; InCHIANTI, Invecchiare in Chianti, Aging in the Chianti Area; IQR, interquartile range; MCSA, Mayo Clinic Study of Aging; MMSE, Mini-Mental State Examination; SNAC-K, The Swedish National Study on Aging and Care in Kungsholmen; SRT, Buschke Selective Reminding Test.

^a^ The initial assessment of 6-m gait speed started in 2006, although the BLSA began in 1958. The sample in the Health ABC is from the Cognitive Vitality Substudy.

^b^ Calculated as weight in kilograms divided by height in meters squared.

^c^ A higher score indicates higher performance. The range of possible scores is 0 to 30 for the MMSE and 0 to 100 for the 3MS.

^d^ The MMSE memory subscore is shown as median (IQR).

^e^ A higher value indicate higher performance.

^f^ Only visits where memory function and gait speed were assessed and used to define the phenotypic classification.

^g^ In AGES-RS, gait and memory were assessed only twice with a mean (SD) interval of 5 (0.3) years.

Compared with usual agers, after adjustment, participants with gait decline and participants with dual decline had faster baseline gait speed in all studies except InCHIANTI. Baseline gait speed did not differ between participants with memory decline and usual agers in all studies (eTable 2 in the Supplement).

Compared with usual agers, participants with memory decline and participants with dual decline had higher baseline memory performance in all studies except InCHIANTI and BLSA. Baseline memory did not differ between participants with gait decline and usual agers except in Health ABC, in which participants with gait decline had poorer baseline memory performance (eTable 2 in the Supplement).

After adjustment, poorer baseline memory performance was associated with a higher risk of developing dementia in all studies except InCHIANTI. Slower baseline gait speed was associated with a higher risk of dementia in all studies except BLSA (Table 2; model 1).

**Table 2.** Associations of Phenotypic Groups With Subsequent Dementia Risk^a^

Phenotype	BLSA (N = 664)		Health ABC (N = 727)		MCSA (N = 2633)		AGES-RS (N = 2563)		InCHIANTI (N = 553)		SNAC-K (N = 1559)	
	HR (95% CI)	*P* Value	HR (95% CI)	*P* Value	HR (95% CI)	*P* Value	HR (95% CI)	*P* Value	HR (95% CI)	*P* Value	HR (95% CI)	*P* Value
Model 1												
Baseline memory	0.951 (0.926-0.977)	<.001	0.983 (0.966-1.000)	.04	0.942 (0.917-0.969)	<.001	0.946 (0.928-0.965)	<.001	1.008 (0.836-1.215)	.93	0.923 (0.867-0.982)	.01
Baseline gait speed, m/s	1.270 (0.261-6.190)	.76	0.259 (0.104-0.643)	.004	0.228 (0.080-0.650)	.005	0.293 (0.135-0.633)	.001	0.279 (0.106-0.734)	.009	0.440 (0.236-0.818)	.009
Model 2												
No memory or gait speed decline	[Reference]	NA	[Reference]	NA	[Reference]	NA	[Reference]	NA	[Reference]	NA	[Reference]	NA
Memory decline only	4.473 (2.036-9.826)	<.001	4.323 (2.786-6.709)	<.001	4.362 (2.603-7.310)	<.001	2.619 (1.965-3.492)	<.001	2.252 (1.478-3.432)	<.001	4.633 (2.916-7.359)	<.001
Gait decline only	2.418 (0.789-7.411)	.12	2.166 (1.108-4.233)	.02	1.889 (0.915-3.902)	.08	1.558 (0.945-2.568)	.08	3.629 (1.971-6.683)	<.001	2.503 (1.564-4.006)	<.001
Dual decline in memory and gait speed	5.912 (2.252-15.524)	<.001	11.722 (6.346-21.651)	<.001	5.810 (3.040-11.104)	<.001	5.206 (3.484-7.781)	<.001	6.993 (3.654-13.385)	<.001	5.332 (3.166-8.982)	<.001
Baseline memory	0.932 (0.904-0.960)	<.001	0.957 (0.939-0.975)	<.001	0.928 (0.903-0.955)	<.001	0.930 (0.911-0.949)	<.001	1.030 (0.856-1.239)	.75	0.849 (0.797-0.904)	<.001
Baseline gait speed, m/s	0.997 (0.200-4.965)	.99	0.196 (0.077-0.503)	<.001	0.183 (0.063-0.536)	.001	0.241 (0.109-0.533)	<.001	0.200 (0.078-0.513)	<.001	0.302 (0.150-0.605)	<.001

Abbreviations: AGES-RS, Age, Gene/Environment Susceptibility-Reykjavik Study; BLSA, Baltimore Longitudinal Study of Aging; Health ABC, Health, Aging and Body Composition Study; HR, hazard ratio; InCHIANTI, Invecchiare in Chianti, Aging in the Chianti Area; MCSA, Mayo Clinic Study of Aging; NA, not applicable; SNAC-K, The Swedish National Study on Aging and Care in Kungsholmen.

^a^ All models were adjusted for baseline age, sex, and educational level in all studies; additionally adjusted for race/ethnicity in BLSA, Health ABC, and MCSA; and additionally adjusted for site in Health ABC and InCHIANTI.

Compared with usual agers, participants with dual decline and participants with memory decline had a significantly higher risk of developing dementia consistently across all studies after adjustment for demographic characteristics and baseline memory and gait speed (Table 2; model 2; eFigure in the Supplement). Specifically, participants with dual decline had 5.2 to 11.7 times higher risk of developing dementia compared with usual agers, with a pooled hazard ratio (HR) of 6.28 (95% CI, 4.56-8.64) (Figure, C).^15,16,17,18,19,20^ Participants with only memory decline had 2.2 to 4.6 times higher risk, with a pooled HR of 3.45 (95% CI, 2.45-4.86) (Figure, A).^15,16,17,18,19,20^ Participants with only gait decline had 2.1 to 3.6 times the risk (pooled HR, 2.24; 95% CI, 1.62-3.09) (Figure, B).^15,16,17,18,19,20^ Associations of gait decline with dementia risk were significant in Health ABC, InCHIANTI, and SNAC-K but not significant in BLSA, MCSA, and AGES-RS. Meta-analyses results remained similar after excluding 2 studies in which dementia was ascertained a posteriori: compared with usual agers, participants with dual decline had a pooled HR of 5.40 (95% CI, 4.95-5.89), participants with only memory decline had a pooled HR of 3.62 (95% CI, 2.22-5.90), and participants with only gait decline had a pooled HR of 2.01 (95% CI, 1.36-2.98). Adjustment for multimorbidity and specific diseases did not substantially alter the results (eTable 3 in the Supplement).

**Figure.**
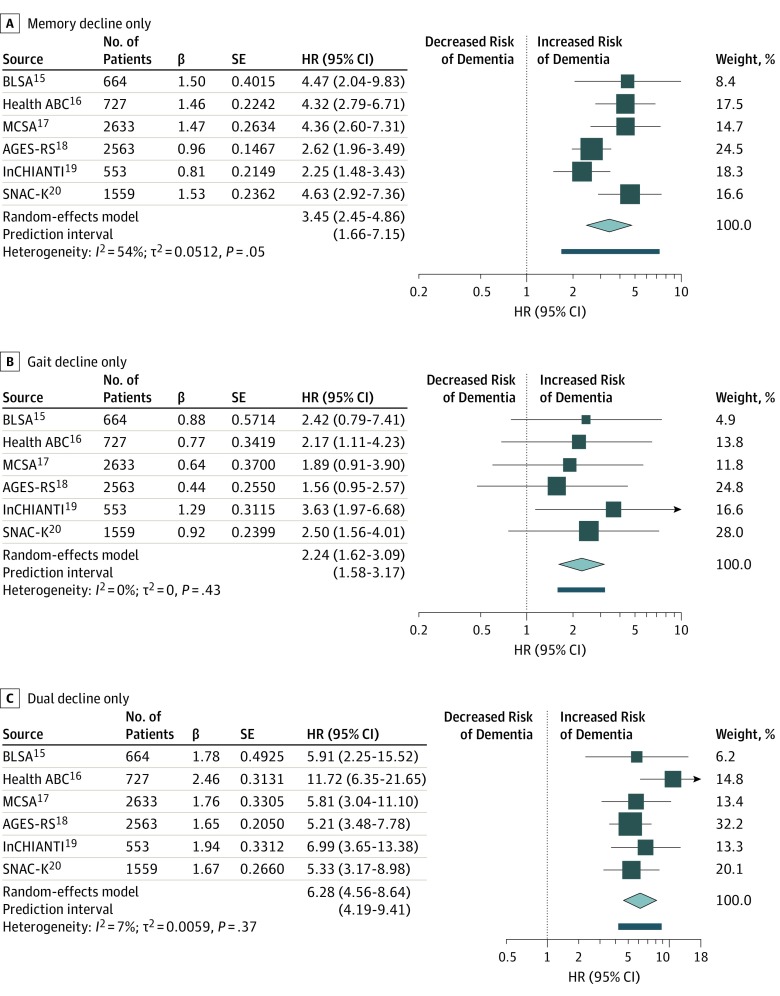
Forest Plots of Hazard Ratios (HRs) for Dementia Risk in Phenotypic Groups A, Participants with only memory decline compared with those with no decline in memory or gait speed. B, Participants with gait decline only compared with those with no decline in memory or gait speed. C, Participants with dual decline compared with those with no decline in memory or gait speed. The sizes of the data markers indicate the size of each study; the larger the data marker, the more participants were in the study. AGES-RS indicates the Age, Gene/Environment Susceptibility-Reykjavik Study; BLSA, the Baltimore Longitudinal Study of Aging; Health ABC, the Health, Aging and Body Composition Study; InCHIANTI, Invecchiare in Chianti, Aging in the Chianti Area; MCSA, the Mayo Clinic Study of Aging; and SNAC-K, the Swedish National Study on Aging and Care-Kungsholmen Population Study. SE indicates standard error.

Based on likelihood ratio tests performed consistently across the 6 studies, models including information on change over time yielded a lower Akaike information criterion and a significant model change compared with models including information on baseline gait speed and baseline memory only, indicating a better fit (eTable 4 in the Supplement).

In BLSA, Health ABC, and SNAC-K, the interaction between slope of memory decline and slope of gait decline was significant in Cox proportional hazards regression models associated with dementia risk (BLSA: β [SE], –1.285 [0.495]; *P* = .01; Health ABC: β [SE], –3.071 [1.137]; *P* = .006; and SNAC-K: β [SE], –7.590 [2.403]; *P* = .001). In MCSA, findings were not significant (β [SE], 0.595 [0.362]; *P* = .10). In AGES-RS and InCHIANTI, the interaction term was also not significant (AGES-RS: β [SE], 2.10 [1.51]; *P* = .16; InCHIANTI: β [SE], –0.053 [0.053]; *P* = .31 [in InCHIANTI, the slope of gait and slope of memory (by Poisson) were multiplied by 100 owing to small values]).

## Discussion

Across 6 large cohort studies of aging, dual decline in both memory and gait speed during the dementia-free period was associated with high risk of developing dementia. Our findings are consistent with the recent report by Montero-Odasso and colleagues.^10^ Our data further contribute to the literature by confirming these findings in 6 geographically and culturally diverse aging studies, focusing on memory function, rather than global cognition, and by characterizing early phenotype when participants are free of dismobility, cognitive impairment, and dementia.

Poorer baseline memory was associated with a higher risk of dementia except in InCHIANTI. The lack of an association in InCHIANTI may be due to the use of the MMSE memory subscore, a poorly sensitive measure of memory. In all studies except BLSA, slower baseline gait speed was associated with a higher risk of dementia, consistent with prior findings.^1^ The lack of an association in BLSA supports the notion that a 1-time gait speed assessment may be less able than change over time to assess risk of dementia.^10^ In all 6 studies, models with change over time significantly improved the assessment of the risk of dementia than models with only information on baseline gait speed and memory. Thus, tracking changes over time may add important information in assessing the risk of developing dementia beyond obtaining a 1-time assessment.

As expected, participants with memory decline had an increased risk of dementia consistently across the 6 cohorts. Our focus on verbal episodic memory, especially immediate recall, rather than global cognition or mental status, allows us to capture early memory decline among Alzheimer disease–related cognitive measures within aging. Focusing on memory, rather than executive function or processing speed, also allows us to better differentiate phenotypic groups because gait speed is associated with executive function within cognitive domains.^42^

Although participants with gait decline also showed some increased risk of dementia, when accompanied by a parallel decline in objective memory, the risk of developing dementia tended to be the highest among phenotypic groups. Our finding that participants with dual decline are at particularly high risk of developing dementia supports the importance of the unique occurring symptom of cognitive and motor deficits outlined in previous research on motoric cognitive risk syndrome and further establishes its clinical relevance. Among older adults living in the community free of cognitive impairment and dismobility, the dual decline phenotype can be captured early in clinical settings by routinely administering gait speed assessment and a free recall memory test.^43^ At this stage, this group should be carefully evaluated for potentially reversible risk factors for dementia. However, research focusing on biological and physiological characteristics that explain why individual with dual decline are at such high risk of developing dementia may lead to new opportunities for prevention.

Our findings add weight to the hypothesis that dual decline with aging represents some shared underlying processes associated with declines in both gait and memory that are associated with ultimate overt dementia. Our study may provide a possible explanation for the heterogeneity of reported findings.^1,3^ Perhaps gait speed decline is associated with dementia most particularly when it is at least in part due to incipient central nervous system pathologic characteristics. Our findings that participants with dual decline have the highest risk of dementia capture the critical need for tracking gait and memory and may help isolate potential central nervous system causes of gait decline. An interesting hypothesis is that dual decline may characterize older persons who will eventually progress to dementia through specific pathophysiological mechanisms. Although the nature of these mechanisms is not yet known, we propose that vascular, metabolic, or energetic dysfunction is the likely factor.^44,45,46,47,48,49,50,51,52,53,54,55^ This hypothesis should be tested in further studies by examining the particular metabolic, vascular, and neuroimaging features that characterize this specific group.

Our findings do not necessarily address the question as to whether the high risk of dementia associated with dual decline is simply due to the combination of 2 independent factors, gait decline and memory decline, or whether the increased risk represents a shared underlying pathologic condition. Findings varied across the 6 cohorts. Specifically, while the relative risk of dementia associated with dual decline was always higher than the sum of risks due to gait decline and memory decline, the interaction between gait decline and memory decline was significant in 3 studies (BLSA, Health ABC, and SNAC-K). In studies that demonstrated a significant interaction, the period in which gait and memory were assessed was relatively long (mean period, 6-9 years) vs AGES-RS and MCSA (5 years). Although InCHIANTI also had a relatively long period of time assessing gait and memory (9 years), it was somewhat limited by the insensitive measure of memory, based only on the MMSE memory subscore.

### Strengths and Limitations

This study has numerous strengths: use of multiple large longitudinal cohort studies, efforts to harmonize measures, careful delineation of individual study approaches to dementia diagnosis, and use of rigorous meta-analytic measures. Accounting for multimorbidity and specific diseases, such as hypertension, cardiovascular disease, and stroke, enriched the analyses, and the findings remained robust. Although a posteriori ascertainment of dementia in 2 studies may have overestimated incident cases, the results remained largely unchanged after excluding these 2 studies.

This study also has some limitations. We acknowledge a lack of information on subtypes of dementia in some studies; thus, we could not evaluate the risk of dementia subtypes. Second, the specific memory measures that we examined in InCHIANTI and MCSA are partly used in dementia diagnostic procedures. However, in InCHIANTI, the MMSE is not the only assessment used to ascertain dementia, and our study used the memory subscore. In MCSA, information from the Rey Verbal Learning Test among all other neuropsychological tests contributed to the determination of a dementia diagnosis.^17^ In the other 4 studies, the memory measures that we used in this study are not used in their dementia diagnostic algorithms. Another potential limitation is the possibility of regression to the mean. In some studies, participants with dual decline had higher baseline gait speed and memory performance than usual agers. Participants with dual decline who had higher baseline gait and memory may have had more room to decline than those with lower baseline performance. However, by adjusting for baseline gait speed and memory, we minimized this possibility.

## Conclusions

Our findings have relevant clinical implications and provide important contributions to research on early signs of an underlying dementia process. Older persons with dual decline in memory and gait speed should receive further attention to address issues that may increase dementia risk, including evaluation of cardiovascular and metabolic risk factors. Studying the cardiovascular and metabolic characteristic of this subgroup may provide clues to the specific mechanisms that confer high risk of dementia. Dual decline in memory and gait speed was associated with high risk of developing dementia. These individuals might serve as a valuable target for preventive interventions. Why individual with dual decline are at such a high risk of developing dementia and whether they progress to dementia through distinct mechanisms are not yet known and deserve further investigation.

## Notes

Supplement. **eFigure.** Kaplan-Meier Survival Curve Between Phenotypic Groups and Dementia Risk **eTable 1.** Study Design and Detailed Assessment **eTable 2.** Associations of Baseline Gait Speed and Baseline Memory Performance With Phenotypic Groups **eTable 3.** Associations of Phenotypic Groups With Dementia Risk After Adjustment for Disease Conditions **eTable 4.** Likelihood Ratio Estimates for Model Comparisons **eReferences.**
