# Errors in Figure

**DOI:** 10.1001/jamanetworkopen.2020.4872

**Published:** 2020-04-03

In the Original Investigation titled “Association of Dual Decline in Memory and Gait Speed With Risk for Dementia Among Adults Older Than 60 Years: A Multicohort Individual-Level Meta-analysis,”^1^ published February 21, 2020, there were errors in the forest plots of the Figure. The vertical dashed line indicating the overall measure of effect was missing from each panel, and the 95% CIs in the plot in panel B did not match the actual 95% CIs listed to the left of the plot. This article has been corrected.^1^
