# Supplementary material for: Association of Dual Decline in Memory and Gait Speed With Risk for Dementia Among Adults Older Than 60 Years: A Multicohort Individual-Level Meta-analysis
Source: JAMA Netw Open. 2020 Feb 21;3(2):e1921636. doi: 10.1001/jamanetworkopen.2019.21636 (PMC7043189; doi:10.1001/jamanetworkopen.2019.21636)
Supplement: Supplement. — eFigure. Kaplan-Meier Survival Curve Between Phenotypic Groups and Dementia Risk eTable 1. Study Design and Detailed Assessment eTable 2. Associations of Baseline Gait Speed and Baseline Memory Performance With Phenotypic Groups eTable 3. Associations of Phenotypic Groups With Dementia Risk After Adjustment for Disease Conditions eTable 4. Likelihood Ratio Estimates for Model Comparisons eReferences. [file jamanetwopen-3-e1921636-s001.pdf]

Supplementary Online Content

Tian Q, Resnick SM, Mielke MM, et al. Association of dual decline in memory and gait speed with risk for dementia among adults older than 60 years: a multicohort individual-level meta-analysis. *JAMA Netw Open.* 2020;3(2):e1921636.
doi:10.1001/jamanetworkopen.2019.21636

eFigure. Kaplan-Meier Survival Curve Between Phenotypic Groups and Dementia Risk
eTable 1. Study Design and Detailed Assessment
eTable 2. Associations of Baseline Gait Speed and Baseline Memory Performance With Phenotypic Groups
eTable 3. Associations of Phenotypic Groups With Dementia Risk After Adjustment for Disease Conditions
eTable 4. Likelihood Ratio Estimates for Model Comparisons
eReferences.

This supplementary material has been provided by the authors to give readers additional information about their work.

© 2020 Tian Q et al. *JAMA Network Open.*

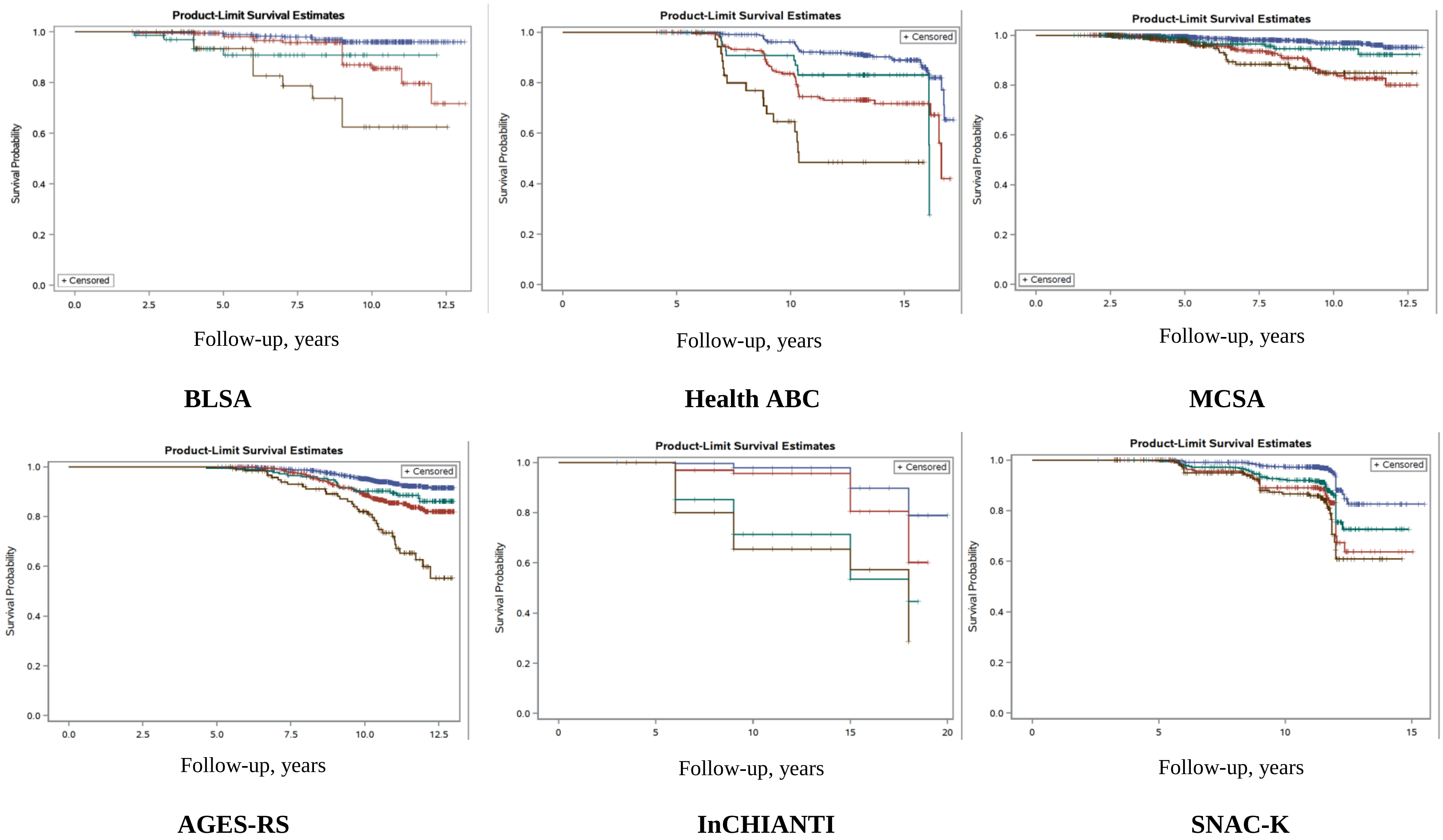

eFigure. Kaplan-Meier Survival Curve Between Phenotypic Groups and Dementia Risk

Group 0 (blue): usual agers. Group 1 (red): memory decliners only. Group 2 (green): gait decliners only. Group 3 (brown): dual decliners

© 2020 Tian Q et al. *JAMA Network Open.*

eTable 1. Study Design and Detailed Assessment

Assessment	BLSA	Health ABC	MCSA	AGES-RS	InCHIANTI	SNAC-K
**Age at study entry**	20+	70-79	30+	65+	65+	60+
**Follow-up interval**	Every 4 years for <60; every 2 years for 60-79; annual for 80+	annual	Every 15 months for those aged 50+; every 30 months for those aged 30-49	5 years apart	Years 3, 6, 9 15, 18	Every 6 years until age 78 and every 3 years after 78
**Study entry**	[a] 2006-	1997/98	2004-	2002	1998-	2001-
**Gait speed**	6m	6m or 20m	25 ft (7.62m)	6m	4m or 7m	2.44m or 6m
**Memory**	**CVLT[1]** (Trial 1-5)<br><br>Range of possible scores: 0-80	**Selective Reminding Test [2]** (Trial 1-5)<br><br>Range of possible scores: 0-72	**AVLT[3]** (Trial 1-5)<br><br>Range of possible scores: 0-75	**Modified CVLT[1]** (Trial 1-4)<br><br>Range of possible scores: 0-64	**MMSE memory subscore[4]**<br><br>Range of possible scores: 0-6	**Word recall test**<br><br>Range of possible scores: 0-16
**Dementia diagnosis**	DSM III-R[5]	(1) 3MS decline (2) medication (3) hospital records[6]	DSM-IV criteria[7]	Three-step procedure following DSM-IV criteria[8]	[b] Two-step screening procedure following DSM-IV criteria [9]	DSM-IV criteria[10]

Note. CVLT=California Verbal Learning Test. AVLT=Auditory Verbal Learning Test. MMSE=Mini-mental state exam. [a] The initial 6m gait speed assessment in the BLSA. [b] a panel of geriatricians and investigators who established the diagnosis of dementia in InCHIANTI followed DSM-IV criteria.

© 2020 Tian Q et al. *JAMA Network Open.*

eTable 2. Associations of Baseline Gait Speed and Baseline Memory Performance With Phenotypic Groups

Phenotypic groups	BLSA (n=664)	Health ABC (n=727)	MCSA (n=2633)	AGES-RS (n=2563)	InCHIANTI (n=553)	SNAC-K (n=1559)
	**baseline gait speed, m/sec β (SE), p-value**					
**Usual agers (reference)**	-	-	-	-	-	-
**Memory decliners only**	-0.027 (0.017) 0.11	-0.017 (0.018) 0.34	-0.016 (0.010) 0.11	0.005 (0.007) 0.47	-0.007 (0.018) 0.67	-0.013 (0.016) 0.39
**Gait decliners only**	0.134 (0.025) <0.001	0.062 (0.028) 0.03	0.146 (0.012) <0.001	0.153 (0.013) <0.001	0.050 (0.027) 0.06	0.200 (0.015) <0.001
**Dual decliners**	0.075 (0.028) 0.009	0.071 (0.035) 0.04	0.156 (0.014) <0.001	0.110 (0.015) <0.001	0.066 (0.035) 0.06	0.176 (0.018) <0.001
	**baseline memory β (SE), p-value**					
**Usual agers (reference)**	-	-	-	-	-	-
**Memory decliners only**	5.824 (1.021) <0.001	8.279 (0.833) <0.001	3.567 (0.372) <0.001	3.773 (0.280) <0.001	-0.047 (0.094) 0.61	1.848 (0.137) <0.001
**Gait decliners only**	-3.054 (1.479) 0.03	-2.796 (1.322) 0.03	-0.552 (0.449) 0.21	-0.466 (0.496) 0.34	0.174 (0.139) 0.21	0.104 (0.130) 0.42
**Dual decliners**	1.910 (1.698) 0.26	6.055 (1.609) <0.001	2.813 (0.522) <0.001	2.830 (0.587) <0.001	-0.179 (0.180) 0.32	1.775 (0.160) <0.001

Note. All models were adjusted for baseline age, sex, education in all studies, additionally adjusted for race in BLSA, Health ABC, and MCSA, and additionally site in Health ABC and InCHIANTI.

eTable 3. Associations of Phenotypic Groups With Dementia Risk After Adjustment for Disease Conditions

Model	Phenotypic groups	BLSA (n=637)	Health ABC (n=727)	MCSA (n=2633)	AGES-RS (n=2563)	InCHIANTI (n=553)	SNAC-K (n=1559)
		HR (95% CI), p-value					
Model 1: adjust for multimorbidity	Usual agers (reference)	-	-	-	-	-	-
	Memory decliners only	4.258 (1.895, 9.568) <0.001	4.357 (2.805, 6.769) <0.001	4.368 (2.606, 7.321) <0.001	2.622 (1.967, 3.495) <0.001	2.260 (1.483, 3.444) <0.001	4.652 (2.928, 7.392) <0.001
	Gait decliners only	2.038 (0.603, 6.894) 0.25	2.101 (1.073, 4.113) 0.03	1.915 (0.925, 3.962) 0.08	1.552 (0.941, 2.559) 0.08	3.638 (1.968, 6.725) <0.001	2.556 (1.595, 4.094) <0.001
	Dual decliners	6.130 (2.298, 16.352) <0.001	11.530 (6.237, 21.316) <0.001	5.854 (3.063, 11.187) <0.001	5.198 (3.477, 7.771) <0.001	6.915 (3.572, 13.388) <0.001	5.436 (3.222, 9.170) <0.001
Model 2: Model 1 + specific diseases	Usual agers (reference)	-	-	-	-	-	-
	Memory decliners only	4.332 (1.924, 9.751) <0.001	4.385 (2.820, 6.918) <0.001	4.333 (2.583, 7.269) <0.001	2.624 (1.967, 3.500) <0.001	2.284 (1.494, 3.490) <0.001	4.654 (2.929, 7.395) <0.001
	Gait decliners only	2.558 (0.745, 8.786) 0.13	2.143 (1.090, 4.215) 0.02	1.925 (0.930, 3.986) 0.07	1.553 (0.941, 2.561) 0.08	3.698 (1.991, 6.870) <0.001	2.557 (1.596, 4.096) <0.001
	Dual decliners	6.372 (2.386, 17.022) <0.001	11.429 (6.178, 21.142) <0.001	5.830 (3.047, 11.153) <0.001	5.204 (3.478, 7.786) <0.001	6.984 (3.596, 13.561) <0.001	5.441 (3.224, 9.182) <0.001

Note. All models adjusted for baseline age, sex, education, baseline gait speed and memory performance in all studies, additionally adjusted for race in BLSA, Health ABC, and MCSA, and additionally adjusted for study site in Health ABC and InCHIANTI. The sample size in BLSA differed from Table 1 is due to available data on multimorbidity and specific diseases. Specific diseases important for gait and memory decline include hypertension, stroke, and cardiovascular disease.

© 2020 Tian Q et al. *JAMA Network Open.*

eTable 4. Likelihood Ratio Estimates for Model Comparisons

Model	BLSA (n=664)	Health ABC (n=727)	MCSA (n=2633)	AGES-RS (n=2563)	InCHIANTI (n=553)	SNAC-K (n=1559)
	**AIC (lower value, better model fit)**					
**Model 1: Baseline gait speed, Baseline memory**	499.7	1485.4	1240.4	3647.8	1357.3	2049.5
**Model 2: Baseline gait speed, Baseline memory, + phenotypic groups**	486.3	1422.3	1202.1	3579.0	1324.9	1996.8
	**Chi-square value (p-value)**					
**Model 2 vs. Model 1 Maximal likelihood test, p-value**	19.353 (<0.001)	69.11 (<0.001)	44.282 (<0.001)	74.849 (<0.001)	38.404 (<0.001)	58.728 (<0.001)

Note. All models were adjusted for baseline age, sex, education in all studies, additionally adjusted for race in BLSA, Health ABC, and MCSA, and additionally study site in Health ABC and InCHIANTI.

© 2020 Tian Q et al. *JAMA Network Open.*

eReferences

1. Delis DC, Kramer JH, Kaplan E, Ober BA. *California Verbal Learning Test.* Research ed. New York, NY: Psychological Corporation; 1987.
2. Buschke H, Fuld PA. Evaluating storage, retention, and retrieval in disordered memory and learning. *Neurology.* 1974;24(11):1019-1025.
3. Rey A. *Auditory-verbal learning test (AVLT).* Press Universitaire de France; 1964.
4. Tombaugh TN, McIntyre NJ. The mini-mental state examination: a comprehensive review. *J Am Geriatr Soc.* 1992;40(9):922-935.
5. Association AP. *Diagnostic and statistical manual of mental disorders.* 3rd ed. Washington, DC: American Psychiatric Association; 1987.
6. Hong CH, Falvey C, Harris TB, et al. Anemia and risk of dementia in older adults: findings from the Health ABC study. *Neurology.* 2013;81(6):528-533.
7. Roberts RO, Geda YE, Knopman DS, et al. The Mayo Clinic Study of Aging: design and sampling, participation, baseline measures and sample characteristics. *Neuroepidemiology.* 2008;30(1):58-69.
8. Harris TB, Launer LJ, Eiriksdottir G, et al. Age, Gene/Environment Susceptibility-Reykjavik Study: multidisciplinary applied phenomics. *Am J Epidemiol.* 2007;165(9):1076-1087.
9. Cherubini A, Martin A, Andres-Lacueva C, et al. Vitamin E levels, cognitive impairment and dementia in older persons: the InCHIANTI study. *Neurobiol Aging.* 2005;26(7):987-994.
10. Laukka EJ, Lovden M, Herlitz A, et al. Genetic effects on old-age cognitive functioning: a population-based study. *Psychology and aging.* 2013;28(1):262-274.

© 2020 Tian Q et al. *JAMA Network Open.*

11. Stroup DF, Berlin JA, Morton SC, et al. Meta-analysis of observational studies in epidemiology: a proposal for reporting. Meta-analysis Of Observational Studies in Epidemiology (MOOSE) group. *JAMA.* 2000;283(15):2008-2012.

© 2020 Tian Q et al. *JAMA Network Open.*
